# Methodological considerations of evaluating the rate of presynaptic dopaminergic denervation in Parkinson disease with radiotracers: Analysis of the PPMI data

**DOI:** 10.1097/MD.0000000000026534

**Published:** 2021-07-02

**Authors:** Jeong Won Lee, Yoo Sung Song, Hyeyun Kim, Bon D. Ku, Won Woo Lee

**Affiliations:** a Department of Nuclear Medicine, Catholic Kwandong University College of Medicine, International St. Mary's Hospital, Incheon, Republic of Korea; b Department of Nuclear Medicine, Seoul National University Bundang Hospital, Seongnam, Republic of Korea; c Department of Neurology, Catholic Kwandong University College of Medicine, International St. Mary's Hospital, Incheon, Republic of Korea; d Institute of Radiation Medicine, Medical Research Center, Seoul National University, Seoul, Republic of Korea.

**Keywords:** dopaminergic denervation, Parkinson disease, Parkinson Progression Markers Initiative

## Abstract

Many previous studies have estimated the rate of dopaminergic denervation in Parkinson disease (PD) via imaging studies. However, they lack the considerations of onset age, disease duration at onset, gender, and dopaminergic denervation due to normal aging. Herein, using a large prospective cohort, we estimated the rate of dopaminergic denervation in PD patients, compared with an age- and gender-matched normal control group.

One hundred forty-one normal controls and 301 PD patients were enrolled. Striatal specific binding ratios (SBRs) of I-123 FP-CIT single positron emission tomography images were analyzed according to the age of onset, gender, and the duration of motor symptoms.

In the PD group, symptom duration was significantly correlated with caudate SBRs, but with putamen SBRs (*P* *<* .05, *R*^2^ = 0.02). Moreover, was significantly inversely related to caudate SBRs, but not with putamen SBRs (*P* *<* .05, *R*^2^ = 0.02). Patients of different age onsets did not show any significant correlation between symptom durations and striatal SBRs. In the age-matched group, no significant relationship was observed between symptom duration and percent decrease of caudate SBRs, but there was a significant relationship between symptom duration and percent decrease of the putamen SBRs (*P* *<* .01, *R*^2^ = 0.06). There was no significant relationship between the symptom duration and the percent decrease of striatal SBRs in the age- and gender-matched group.

The significance and *R*^2^ values from the regression analysis between symptom duration, age, and dopaminergic denervation are low. This suggests that, contrary to previous knowledge, there is a relatively weak association between dopaminergic denervation and age or symptom duration.

## 1 Introduction

Parkinson disease (PD) is a neurodegenerative disorder that presents heterogenous symptoms with a highly variable disease progression rate.^[1,2]^ Most of its related symptoms are caused by denervation of the nigrostriatal dopaminergic pathway, which is a lifelong neurodegenerative process.^[3]^ There is evidence supporting the hypothesis that the progression of pathologic process precedes the development of motor related symptoms, even up to decades.^[4]^ For example, several autonomic features, such as constipation and REM sleep behavior disorders (RBD), may appear 20 years before the onset of tremor and bradykinesia. Therefore, the diagnosis of PD is not usually made until there are profound loss of nigrostriatal neurons, since it is mainly based on the presented motor features. Previous studies have proposed that 50% to 80% of the dopaminergic neurons are already lost during the prodromal stage. ^[5,6]^ Estimating the duration and degree of this dopaminergic denervation during the disease progression is important to establish effective treatment strategies. While current treatment options for PD are limited to supplementing dopaminergic tones for symptomatic alleviation, multiple treatment strategies with the potential for disease modification are under investigation. ^[7]^ Based on current information, these disease-modifying treatments are most effective when used during the early stages of the disease, that is, at the initiation of dopaminergic denervation. Thus, numerous studies have attempted to estimate the duration and degree of the nigrostriatal denervation during the progression of PD, by either autopsy or functional imaging studies.

Bernheimer et al first performed a neurochemical correlative autopsy study in patients with Parkinsonism in 1973.^[8]^ They were the first to report that patients with even mild Parkinsonism had a proportionately high degree of dopaminergic denervation; however, they did not perform any regression analysis to determine the relationship between the degree of dopaminergic denervation and duration of disease. Another autopsy study by Riederer et al suggested a 68% to 82% decrease of dopamine concentration in the caudate, compared with the age-matched normal control counterparts.^[9]^ Afterwards, numerous imaging studies have also investigated the degree of dopaminergic denervation at the onset of disease, by measuring L-DOPA metabolism (F-18 FDOPA), dopamine transporter binding (I-123 FP-CIT, I-123 IPT, I-123 β-CIT, etc), and vesicular monoamine transporter binding (C-11 DTBZ), etc (Table 1). These imaging studies have also suggested a substantial loss (20%–70%) of dopaminergic innervation at the onset of motor symptoms. However, these previous studies have several limitations in estimating the degree and duration of dopaminergic neuronal loss. Previous histopathologic studies were done retrospectively, with a small number of patients, and could not be performed in patients of early stage. Moreover, prior imaging studies were also done with a small number of subjects, and/or did not consider gender differences dopaminergic denervation due to normal aging, or symptom duration upon enrollment.

**Table 1:** PD imaging studies of dopaminergic denervation.

Author	Number of PD patients	Ligand	Normal control group (number)	Follow up scan	Analysis	Results
Bohnen et al^[10]^	31	C-11 DTBZ	Yes (75)	No	Striatal BP measurement	Significant correlation of striatal BP decline with symptom duration
de la Fuente-Ferna’ ndez et al^[11]^	78	C-11 DTBZ	Yes (35)	Yes	Exponential curve analysis	Higher rate of DTBZ BP decline in older onset, compared with younger onset PD patients
Morrish et al^[12]^	32	F-18 FDOPA	Yes (16)	Yes	Influx constant (Ki) measurement	4.7% decline per year
Morrish et al^[13]^	10	F-18 FDOPA	Yes (10)	Yes	Influx constant (Ki) measurement	12.5% decline per year
Hiker et al^[14]^	31	F-18 FDOPA	No	Yes	Influx constant (Ki) measurement	4.4% (caudate), 6.3% (putamen) decline per year
Nurmi et al^[15]^	8	F-18 CFT	Yes (7)	Yes	Striatal SBR measurement	12.5% (caudate), 13.1% (putamen) decline per year
Tissingh et al^[16]^	21	I-123 FP-CIT	Yes (14)	No	Striatal SBR measurement	9.6% decline per 10 years of age
Pirker et al^[17]^	36	I-123 β-CIT	No	Yes	Striatal SBR measurement	7.1% decline per year in early stage PD
Chouker et al^[18]^	8	I-123 IPT	Yes (8)	Yes	Striatal SBR measurement	6.6% decline in the first year, 5.3% decline in the second year

PD = Parkinson disease, SBR = specific binding ratio.

The primary goal of our large prospective cohort study was to estimate the progression rate and the degree of dopaminergic denervation in PD patients, and compare them with those in age- and gender-matched normal control group. We analyzed the [I-123] N-ω-fluoropropyl- 2β-carbomethoxy- 3β-(4-iodophenyl) nortropane (I-123 FP-CIT) single photon emission computed tomography (SPECT) images from the Parkinson Progression Markers Initiative (PPMI) database.

## 2 Methods

### 2.1 Patients

**Figure 1:**
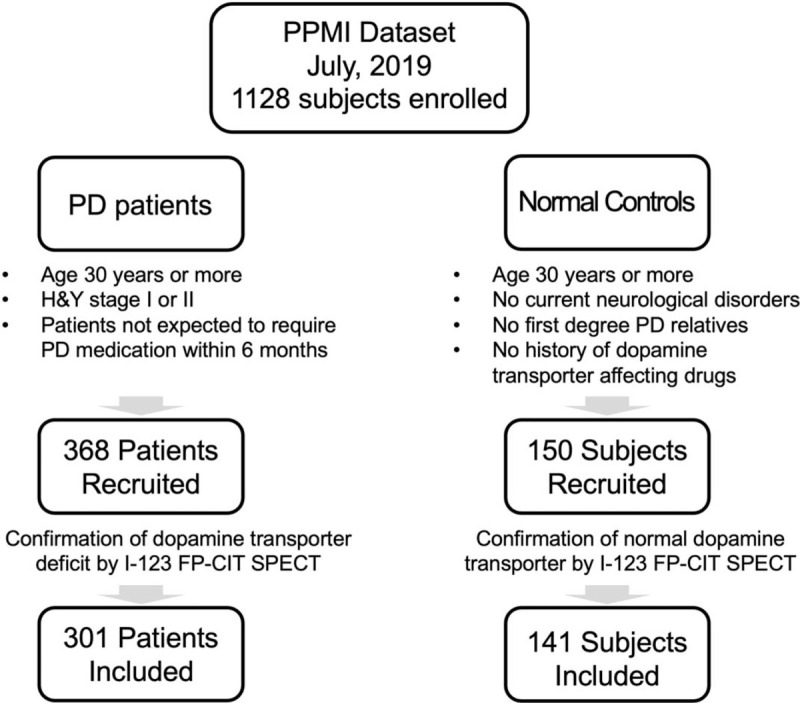
Flow chart of patients reviewed. H&Y = Hoehn and Yahr, I-123 FP-CIT SPEDT = [I-123] N-ω-fluoropropyl- 2β-carbomethoxy- 3β-(4-iodophenyl) single positron emission tomography, PD = Parkinson disease.

Data were downloaded from the Parkinson Progression Markers Initiative (PPMI) database (http://www.ppmi-info.org) in July 2019 (Fig. 1). The PPMI is an ongoing multicenter observational cohort study, containing a full set of clinical, imaging and biological data, with the primary goal of identifying biomarkers for the progression of PD. The PPMI database includes both PD subjects and normal control subjects. The inclusion criteria for the subjects are described in http://www.ppmi-info.org. In short, for PD subjects, male or female patients of age 30 years or older, Hoehn and Yahr (H&Y) stage I or II at baseline, patients with confirmation of dopamine transporter deficit by I-123 FP-CIT SPECT or VMAT-2 PET scans, and patients not expected to require PD medication within 6 months from baseline were enrolled. For normal control subjects, male or female age 30 years or older at screening, without currently active significant neurological disorders, without first degree relatives with PD, and without history of drug administration that could interfere with dopamine transporter imaging were enrolled. Data acquisition of PPMI was performed in accordance with the relevant guidelines and regulations. Written informed consent for all clinical data were obtained of all PPMI participants, and all subjects gave written informed consent in accordance with the 1964 Declaration of Helsinki and its later amendments. The study was approved by all respective Institutional review boards (IRBs, 33 institutions, listed at https://www.ppmi-info.org/about-ppmi/ppmi-clinical-sites).

In our analysis, patients and normal control subjects from the PPMI database without baseline I-123 FP-CIT SPECT scans were excluded; leading to a final number of 141 normal controls (age 60.7 ± 11.1, M: F = 88: 53) and 301 PD patients (age 60.9 ± 9.6, M: F = 197: 104). Data of patients and normal control subjects from the PPMI, such as age, gender, subjects’ weight, I-123 FP-CIT SPECT analysis results, previous motor symptom durations at enrollment, H&Y scores, Movement Disorder Society-Unified Parkinson disease rating scale (MDS-UPDRS) II/III scores, and Scale for Outcomes in Parkinson disease-Autonomic (SCOPA-AUT), were adopted.

### 2.2 I-123 FP-CIT SPECT analysis

I-123 FP-CIT SPECT scans were performed 4 ± 0.5 hours after I-123 FP-CIT injection (111–185 MBq) upon enrollment. Iterative reconstruction, with no filtering was applied. Each institution was required to receive technical setup visits from the core imaging lab team of PPMI. This team also performed quality controls to maintain uniformity in data quality among the multiple institutions. Images were analyzed with the PMOD software (PMOD Technologies, Zurich, Switzerland), and the specific binding ratios (SBRs, (target region/reference region)-1) of each caudate and putamen were acquired with the occipital cortex as a reference tissue. For the healthy control group, minimum SBR values among the bilateral striatal regions were selected; and for the PD group, the dominant side were selected for analysis. To determine the percent decrease of striatal SBRs, ratios of striatal SBRs between PD patients and age-matched normal control patients were acquired.

### 2.3 Statistical analysis

Medcalc version 18.9.1 (MedCalc Software, Belgium) was used for analysis. Demographic factors and striatal SBRs between the groups were compared by one-way ANOVA with Scheffé test for post-hoc, or Kruskal-Wallis test with Conover test for post-hoc. For correlation of SBR values and age or symptom duration, regression analysis was done based on an exponential model (SBR = Ae^-Bt^). Age- and gender-matched groups were brought up by randomization, with a maximum age difference of 2 years. Statistical significance was set at *P* *<* .05.

## 3 Results

### 3.1 Demographic characteristics

The demographic characteristics of the normal control (NC) group (n = 141) and the PD patient group (n = 301) are presented in Table 2. There were no significant differences of age at enrollment, gender, and weight between the 2 groups. The SBR values of the caudate nucleus and the putamen were significantly higher in the control group (*P* *<* .001). The average symptom duration at enrollment, H&Y scores, MDS-UPDRS II scores, MDS-UPDRS III scores, and SCOPA-AUT of the PD group were 21.7 ± 15.9 months, 1.2 ± 0.8, 6.7 ± 4.1, 20.4 ± 8.6, and 8.7 ± 5.6, respectively. The clinical characteristics of the PD group according to the age of onset were analyzed, with subgroups of symptom onset of 50 years of age or less, 51 to 60 years of age, 61 to 70 years of age, and over 70 years of age (Table 3).

**Table 2:** Characteristics of the normal control group and the PD group.

	Normal control (n = 141)	PD (n = 301)	*P-*value
Age at enrollment (yr)	60.7 ± 11.1	60.9 ± 9.6	.79
Gender (Male: Female)	88: 53	197: 104	.53
Weight (kg)	78.5 ± 15.8	81.2 ± 17.2	.11
Caudate nucleus SBRs	2.87 ± 0.60	1.82 ± 0.52	<.001
Putamen SBRs	2.01 ± 0.54	0.68 ± 0.22	<.001

PD = Parkinson disease, SBR = specific binding ratio.

**Table 3:** Characteristics of the PD group according to age of onset.

Age of onset	–50 (n = 63)	51–60 (n = 100)	61–70 (n = 107)	71– (n = 31)	*P-*value
Gender (Male: Female)	37: 13	58: 42	78: 29	24: 7	—
Weight (kg)	80.5 ± 18.2	81.1 ± 16.9	82.9 ± 17.3	77.4 ± 15.7	.46
Symptom duration (months)	26.9 ± 19.5	20.7 ± 13.5	19.4 ± 14.3	21.6 ± 18.2	.06
Caudate nucleus SBRs	1.92 ± 0.52	1.83 ± 0.50	1.79 ± 0.53	1.69 ± 0.53	.20
Putamen SBRs	0.70 ± 0.20	0.65 ± 0.22	0.68 ± 0.23	0.71 ± 0.27	.26
H&Y scores	1.43 ± 0.50^a^	1.48 ± 0.52^a^	1.62 ± 0.51^b^	1.68 ± 0.48^b^	<.05
MDS-UPDRS II scores	6.8 ± 4.2	6.0 ± 3.7	6.9 ± 4.3	7.9 ± 4.1	.10
MDS-UPDRS III scores	18.3 ± 7.4	20.0 ± 9.3	21.5 ± 8.5	21.9 ± 8.6	.08
SCOPA-AUT	7.8 ± 5.5	8.1 ± 5.4	9.3 ± 5.7	10.9 ± 5.6	.09

For a particular variable, values with different superscripts indicate statistically significant difference. MDS-UPDRS = Movement Disorder Society-Unified Parkinson disease rating scale, SCOPA-AUT = Scale for Outcomes in Parkinson disease-Autonomic.

### 3.2 Estimates of dopaminergic denervation in normal controls

Within the NC group, as age increases, regression analysis revealed a significant decrease of the caudate (*P* *<* .001) and putamen SBRs (*P* *<* .001) (Table 4).

**Table 4:** Correlation of age and dopaminergic denervation in normal controls.

	R^2^	Slope	95% C.I. for slope		Intercept	95% CI for intercept		*P-*value
Caudate nucleus SBRs	0.10	-0.002	-0.004	-0.001	0.60	0.52	0.68	*<.*001
Putamen SBRs	0.10	-0.003	-0.005	-0.002	0.50	0.39	0.60	*<.*001

### 3.3 Dopaminergic denervation and symptoms in the PD group

Within the PD group, the caudate SBRs did not show a show a significant correlation with H&Y scores (*P* *=* .14), but did with the MDS-UPDRS II scores (*P* *<* .001), MDS-UPDRS III scores (*P* *<* .001), and SCOPA-AUT (*P* *<* .01). The putamen SBRs did not show a show a significant correlation with H&Y scores (*P* *=* .10) and SCOPA-AUT (*P* = .02), but did with the MDS-UPDRS II scores (*P* *<* .001) and MDS-UPDRS III scores (*P* *<* .01) (Table 5).

**Table 5:** Correlation of dopaminergic denervation and symptom severity in PD.

	*R*^2^	Slope	95% C.I. for slope		Intercept	95% CI for intercept		*P-*value
Caudate SBRs								
H&Y scores								.14
MDS-UPDRS II scores	0.05	-0.007	-0.011	-0.004	0.29	0.26	0.32	<.001
MDS-UPDRS III scores	0.04	-0.003	-0.005	-0.001	0.30	0.26	0.34	<.001
SCOPA-AUT	0.03	-0.004	-0.007	-0.001	0.27	0.25	0.31	<.01
Putamen SBRs								
H&Y scores								.10
MDS-UPDRS II scores	0.06	-0.009	-0.013	-0.004	-0.13	-0.17	-0.10	<.001
MDS-UPDRS III scores	0.05	-0.004	-0.006	-0.002	-0.11	-0.16	-0.07	<.01
SCOPA-AUT								.12

MDS-UPDRS = Movement Disorder Society-Unified Parkinson disease rating scale, SCOPA-AUT = Scale for Outcomes in Parkinson disease-Autonomic.

### 3.4 Estimates of dopaminergic denervation in the PD group

Within the PD group, as the symptom duration increases, the caudate SBRs did not show a show a significant decrease (*P* *=* .24), while the putamen SBRs did (*P* *<* .05). In PD patients with symptom onset of 50 years of age or less (*P* *=* .52), 51 to 60 years of age (*P* *=* .46), 61 to 70 years of age (*P* *=* .35), and over 70 years of age (*P* *=* .52) no significant correlation was observed between caudate SBRs and symptom durations. Furthermore, in PD patients with symptom onset of less than 50 years of age (*P* *=* .52), 51 to 60 years of age (*P* *=* .13), 61 to 70 years of age (*P* *=* .16), and over 70 years of age (*P* *=* .56), there was also no significant relationship between putamen SBRs and symptom durations. While the putamen SBRs showed no change as the age increases, the caudate SBRs showed a significant decrease (*P* *<* .05) (Table 6).

**Table 6:** Correlation of age and symptom duration with dopaminergic denervation in PD.

		*R*^2^	Slope	95% CI for slope		Intercept	95% C.I. for intercept		*P-*value
Symptom duration									
All	Caudate nucleus SBRs								.24
patients	Putamen SBRs	0.02	-0.001	-0.002	0.000	-0.17	-0.20	-0.14	<.05
50 or less	Caudate nucleus SBRs								.52
	Putamen SBRs								.52
51–60	Caudate nucleus SBRs								.46
	Putamen SBRs								.13
61–70	Caudate nucleus SBRs								.35
	Putamen SBRs								.16
71 or	Caudate nucleus SBRs								.52
more	Putamen SBRs								.56
Age									
	Caudate nucleus SBRs	0.02	-0.002	-0.003	0.000	0.35	0.26	0.45	<.05
	Putamen SBRs								0.78

SBR = specific binding ratio.

### 3.5 Dopaminergic denervation rate compared with age and gender matched normal controls

The PD group patients were randomly paired with age-matched, and age/gender-matched normal controls. There was no significant relationship between symptom duration and percent decrease of caudate SBRs in the age-matched group (*P* *=* .13), but there was a significant relationship between the symptom duration and percent decrease of putamen SBRs (*P* *<* .01). There was no significant relationship between symptom duration and percent decrease of caudate SBRs (*P* *=* .32) and putamen (*P* *=* .17) in the age/gender-matched group (Table 7).

**Table 7:** Dopaminergic denervation rate of PD compared with age and gender matched normal controls.

	*R*^2^	Slope	95% C.I. for slope		Intercept	95% CI for intercept		*P-*value
Age matched								
Caudate nucleus SBRs								.13
Putamen SBRs	0.06	-0.003	-0.004	0.001	1.58	1.52	1.63	<.1
Age and gender matched								
Putamen SBRs								.32
Caudate nucleus SBRs								.17

PD = Parkinson disease, SBR = specific binding ratio.

## 4 Discussion

In 2003, Braak proposed a histopathologic staging model for PD, based on the spread of Lewy bodies.^[19]^ Since then, it has been widely accepted as it met the need for clearly defining PD stages – diagnosis, prognosis, prediction, and treatment strategy adjustments. However, the Braak staging model has several limitations; it is based on the postmortem cross-sectional data. Moreover, while the phenotypes during the progression of PD become more complicated, the Braak staging lacks reliability in proposing the corresponding pathologic status with symptoms. Since brain biopsy is not possible in normal clinical situations, many studies have attempted to estimate the rate of dopaminergic decline with imaging biomarkers. ^[11,13,14,20,21]^ However, these imaging studies are not without their limitations. In our study, we analyzed the PPMI cohort, and described the dopaminergic denervation rate and made a comparison with the age- and gender-matched normal controls.

Unlike previous studies that have reported significant correlation of symptom durations and dopaminergic denervation, we did not observe any significant relationships between symptom durations and caudate SBRs; however, with increased symptom duration, there was a significant decrease of putamen SBRs, albeit with very low R^2^ values. Based on a regression analysis, the results were similar in the age- and gender-matched normal control group. Here, we suggest several reasons for the discrepancy.

First, the analysis should consider the exponential pattern of dopaminergic denervation. Some previous imaging studies have calculated the denervation rate based on 2 timepoints of respective individuals with a fixed interval, or have acquired the annual decline by dividing the absolute radioligand of the 2 time points with the follow-up period.^[12–15,18]^ It has been suggested that the dopaminergic denervation occurs in an exponential pattern in accordance with the duration of disease, according to several previous postmortem histopathologic studies.^[20]^ Suppose 2 patients have equal striatal SBR values, but different symptom durations. The patient with a shorter symptom duration would have a higher dopaminergic denervation rate than the patient with a longer symptom duration. Therefore, the analysis shall not compare the dopaminergic images from two certain timepoints. In contrast, Pirker et al compared the annual reduction rate of I-123 β-CIT SPECT scans in the subgroups of PD patients with both short and long symptom durations, with age-matched normal controls. ^[17]^ Similar to our results, they reported no significant correlation between striatal β-CIT binding and disease duration. Moreover, it is proposed that it takes decades from the onset of pathological changes to death, in PD.^[22]^The aggravation of dopaminergic denervation already begins when autonomic dysfunction related symptoms appear, which may be 10 to 20 years before the onset of motor symptoms. So, when the motor symptoms begin to appear, a significant amount of dopaminergic innervation has already been lost. However, most studies have investigated the dopaminergic denervation with relevance to the onset of motor symptoms. Correlation of the dopaminergic denervation rate and the symptom duration from the onset of autonomic dysfunctions might derive different results.

Second, several studies have not considered the effect of aging on dopaminergic denervation.^[12–14]^ It has been suggested that the severity of phenotypes worsens with age in PD patients.^[23]^ Additionally, when PD patients have a similar symptom duration, there is a greater tendency of dopaminergic denervation in patients of older age, albeit with questionable significance. Moreover, it is known that aging is also associated with dopaminergic denervation in normal controls.^[9,24,25]^ While subregional pattern of striatal dopaminergic loss differs between PD patients and normal controls,^[26]^ the effect of natural aging on dopaminergic denervation may be superimposed in PD patients. While there was no significant difference of symptom duration between the subgroups with different age onsets in our analysis, we were unable to find a significant correlation between symptom duration and striatal SBRs in PD patients with different ages of onset. To eliminate the effect of normal aging on dopaminergic denervation, we compared the relative decrease of striatal SBRs with an age-matched normal control group. We found no significant relationship with symptom durations with caudate SBRs, and a significant but very low *R*^2^ value with putamen SBRs.

Finally, we conducted a regression analysis comparing the two groups to eliminate the effect of gender differences. While it has been suggested that the rate of dopaminergic denervation of PD patients is not different between men and women, the physiological dopaminergic level is known to be higher in women. ^[27]^ Moreover, the gender differences also affect the dopaminergic innervation in normal controls. ^[28–30]^ However, we were unable to find any significant relationships between symptom duration and the striatal SBRs in the age- and gender-matched group.

In this study, we estimated the rate of dopaminergic denervation in PD patients, in association with symptom durations and aging, and reviewed the possible confounding factors. Unlike numerous previous studies that have demonstrated the relationship or equation between dopaminergic denervation rate and symptom duration, the regression results were not significant or the *R*^2^ values were very low. Here, we conclude that the dopaminergic denervation rate is not significantly dependent on symptom duration and aging. We suggest several possible reasons for the findings. First, PD is a disease with a wide array of phenotypes with a great degree of unexplainable variations. Due to such heterogeneity, it has been attempted to identify subgroups of PD with homogenous phenotypes. However, the differences of dopaminergic denervation between the subgroups are yet unclear. ^[31]^ In addition to onset age and gender, other unknown and external factors, such as genetic inheritance, should be considered in determining the rate of dopaminergic denervation. Second, it is important to note that patients analyzed here have already manifested motor symptoms. Currently, the Movement disorders Society defines three stages of PD: the preclinical stage (presence of neurodegenerative pathology without clinical symptoms), the prodromal stage (presence of early symptoms or signs, yet insufficient for diagnosis), and the clinical stage (presence of classic motor signs sufficient for diagnosis).^[32]^ A significant portion of the dopaminergic innervation is already lost in the preclinical and prodromal stages.^[5,6]^ Since the dopaminergic denervation occurs in an exponential manner, the slope of the denervation rate would be higher in the preclinical and prodromal stages than in the clinical stage.^[20,33]^ An examination of the association between symptom duration and dopaminergic denervation in PD patients in the preclinical and prodromal stages is warranted to fully understand the pattern of dopaminergic denervation. However, the duration of the preclinical stage may be over 20 years before the onset of motor symptoms,^[22]^ making it challenging to conduct such a prospective study with preclinical, prodromal stage patients.

Our study has some limitations. First, we have not analyzed the correlation between motor severity and dopaminergic denervation. The aim of this study was to review the previous literature and analyze the relationship among symptom duration, age, and the rate of dopaminergic denervation. Second, since the I-123 FP-CIT SPECT images were acquired from multiple institutions, there could be variations of image quality. To maintain uniformity across datasets from the various institutions, the PPMI performs quality control procedures during enrollment of respective institutions. Finally, although I-123 FP-CIT is a presynaptic dopaminergic biomarker, it may not totally reflect the neurodegenerative process. The degree of dopaminergic denervation may vary among different dopamine targeting radioligands.^[33]^

In conclusion, we examined the rate of dopaminergic denervation in PD patients, and analyzed whether it correlated with symptom duration. Though there was a significant correlation between the symptom duration and putamen SBRs, and between the symptom duration and percent decrease of the putamen SBRs, we suggest that the nigrostriatal degeneration involves inherently a greater number of unexplainable variations.

## Author contributions

**Conceptualization:** Jeong Won Lee, Yoo Sung Song.

**Data curation:** Jeong Won Lee, Yoo Sung Song.

**Formal analysis:** Jeong Won Lee, Yoo Sung Song, Hyeyun Kim, Bon D. Ku, Won Woo Lee.

**Funding acquisition:** Yoo Sung Song.

**Methodology:** Jeong Won Lee, Yoo Sung Song, Hyeyun Kim, Bon D. Ku, Won Woo Lee.

**Writing – original draft:** Jeong Won Lee, Yoo Sung Song.
